# Evolution of a Meningothelial Meningioma: From WHO Grade 1 to Anaplastic Grade 3 with Extracranial Metastasis Including Extensive Liver Metastasis

**DOI:** 10.3390/diagnostics14070676

**Published:** 2024-03-22

**Authors:** Christer Ruff, Paula Bombach, Georg Gohla, Till-Karsten Hauser, Frank Paulsen, Nick Farhang, Hans Boesmueller, Rudi Beschorner, Malte Bongers

**Affiliations:** 1 Department of Diagnostic and Interventional Neuroradiology, University Hospital Tübingen, Hoppe-Seyler-Str. 3, 72076 Tübingen, Germany; 2 Department of Neurology and Interdisciplinary Neuro-Oncology, University Hospital Tübingen, Hoppe-Seyler-Str. 3, 72076 Tübingen, Germany; paula.bombach@med.uni-tuebingen.de; 3 Hertie Institute for Clinical Brain Research, Eberhard Karls University Tübingen Center of Neuro-Oncology, Ottfried-Müller-Straße 27, 72076 Tübingen, Germany; 4 Center for Neuro-Oncology, Comprehensive Cancer Center Tübingen-Stuttgart, University Hospital of Tuebingen, Eberhard Karls University of Tübingen, Herrenberger Straße 23, 72070 Tübingen, Germany; 5 Department of Radiation Oncology, University Hospital Tübingen, Hoppe-Seyler-Str. 3, 72076 Tübingen, Germany; 6 Department of Diagnostic and Interventional Radiology, Hanau Hospital GmbH, Leimenstraße 20, 63450 Hanau, Germany; 7 Institute of Pathology and Neuropathology, University Hospital Tuebingen, Liebermeisterstr. 8, 72076 Tübingen, Germany; 8 Department of Neuropathology, Institute of Pathology and Neuropathology, University Hospital Tübingen, Calwerstraße 3, 72076 Tübingen, Germany; 9 Department of Diagnostic and Interventional Radiology, University Hospital Tübingen, Hoppe-Seyler-Str. 3, 72076 Tübingen, Germany; malte.bongers@med.uni-tuebingen.de

**Keywords:** meningioma, metastasis, MRI, computed tomography, ultrasound

## Abstract

A 61-year-old patient was diagnosed with a left-sided falx meningioma. Histopathological analysis following extirpation showed a meningothelial meningioma ZNS WHO grade 1 with sparse mitoses. Over the course of 12 years, the patient received irradiation (54.0 Gy), peptide radio-receptor therapy (^177^Lu-DOMITATE) and targeted therapy (mTOR inhibitor). Follow-up imaging revealed an increased size of the residual tumor. Due to increased liver function parameters, imaging of the liver was performed, showing widespread space-occupying lesions with atypical appearance. Biopsy revealed metastasis of the meningioma, now with 2.7 mitoses/mm^2^, necrosis and homozygous CDKN2A/B deletion, corresponding to an anaplastic CNS meningioma WHO grade 3. A second small meningioma on the left petroclival side has been consistent in size over 12 years. Metastatic meningiomas pose a pertinent clinical challenge due to poor prognosis. The lung, bone, liver and cervical lymph nodes are the most common sites of extracranial metastasis. According to the World Health Organization criteria, the most important predictive factor for recurrence and metastasis is the tumor grade.

The initial findings of the falx meningioma and the follow-up imaging postoperatively and over the longer term are shown in Figure 1A–C. Histopathological analysis following extirpation corresponds to a meningioma grade 1 (Figure 1D).

The lung, bone, liver and cervical lymph nodes are the most common sites of extracranial metastasis [1,2]. In this case, the patient developed bone and extensive liver metastases in the course of the disease (Figure 2A,B). Histopathological analysis following biopsy of one of the liver metastases correspond to a meningioma grade 3 (Figure 2C). According to the World Health Organization criteria, the most important predictive factor for recurrence and metastasis is the tumor grade (as mentioned above). Until the new WHO classification of 2021 for central nervous system tumours, predictive factors included, among others, high cellularity, the presence of foci of necrosis and mitotic rate [3]. These additional factors are no longer mentioned in the 2021 WHO classification. The presence of these features is not essential for the occurrence of extracranial metastasis, like in this case with initial WHO grade 1 meningioma [4]

## Figures and Tables

**Figure 1:**
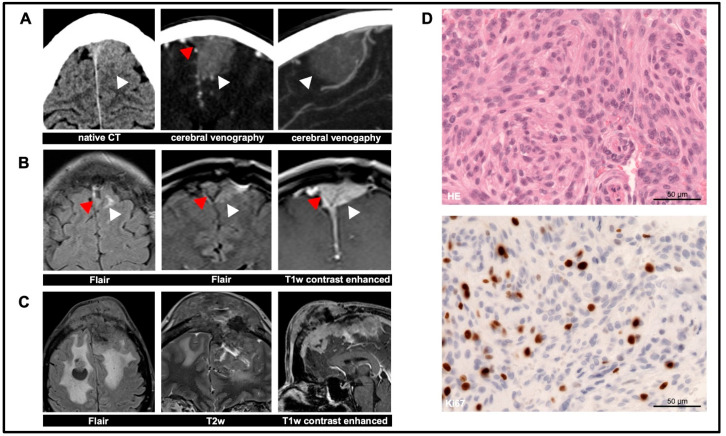
Serial imaging in a patient with a meningioma: (**A**) Initial imaging showing a well-circumscribed extra-axial lesion in the sense of a left-sided falx meningioma without calcification (white arrowhead). Infiltration of the sinus sagittalis superior is present (red arrowhead in cerebral venography). (**B**) Follow-up MRI showing an increase in size of the residual tumor with known infiltration of the superior sagittal sinus (red arrowhead). The images shown were acquired 1.5 years after partial resection. However, the further progression of the residual meningioma was slow in a time frame of 8 years without clinical impact on the patient. (**C**) In the years that followed, there was significant growth in the size of the meningioma despite the therapies mentioned above that were introduced in the meantime, such as peptide radio-receptor therapy (^177^Lu-DOMITATE), targeted therapy (mTOR inhibitor) and re-irradiation. Twelve years after initial partial resection, the meningioma shows extensive space-occupying growth per continuitatem through the calvaria. (**D**) Histopathological images of the primary meningioma (HE, Ki67). FLAIR = fluid-attenuated inversion recovery; T1w = T1-weighted; T2w = T2-weighted.

**Figure 2:**
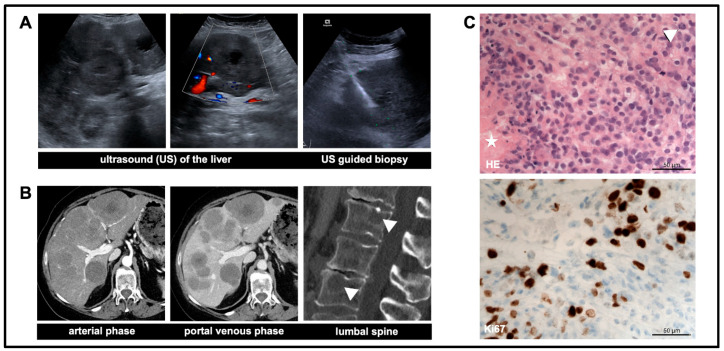
Ultrasound and abdominal computed tomography (CT): (**A**) Ultrasound showing a permeation of almost the entire liver with masses (partly centrally hypoechogenic and not hyperperfused). (**B**) CT confirming the diagnosis showing multiple, extensive metastatic liver lesions in all segments. Additionally, multiple suspicious osteolyses of the entire axial skeleton are detected (white arrowhead). (**C**) Histopathological images of a liver metastasis (HE, Ki6). Note: mitosis (white arrowhead) and necrosis (white asterisk).

## Data Availability

All relevant data are contained within the article.
